# Supplementary figures and images for: Schrödinger’s yeast: the challenge of using transformation to compare fitness among Saccharomyces cerevisiae that differ in ploidy or zygosity
Source: PeerJ. 2023 Dec 5;11:e16547. doi: 10.7717/peerj.16547 (PMC10704993; doi:10.7717/peerj.16547)

resid. Max slope November

control MA petite exclude

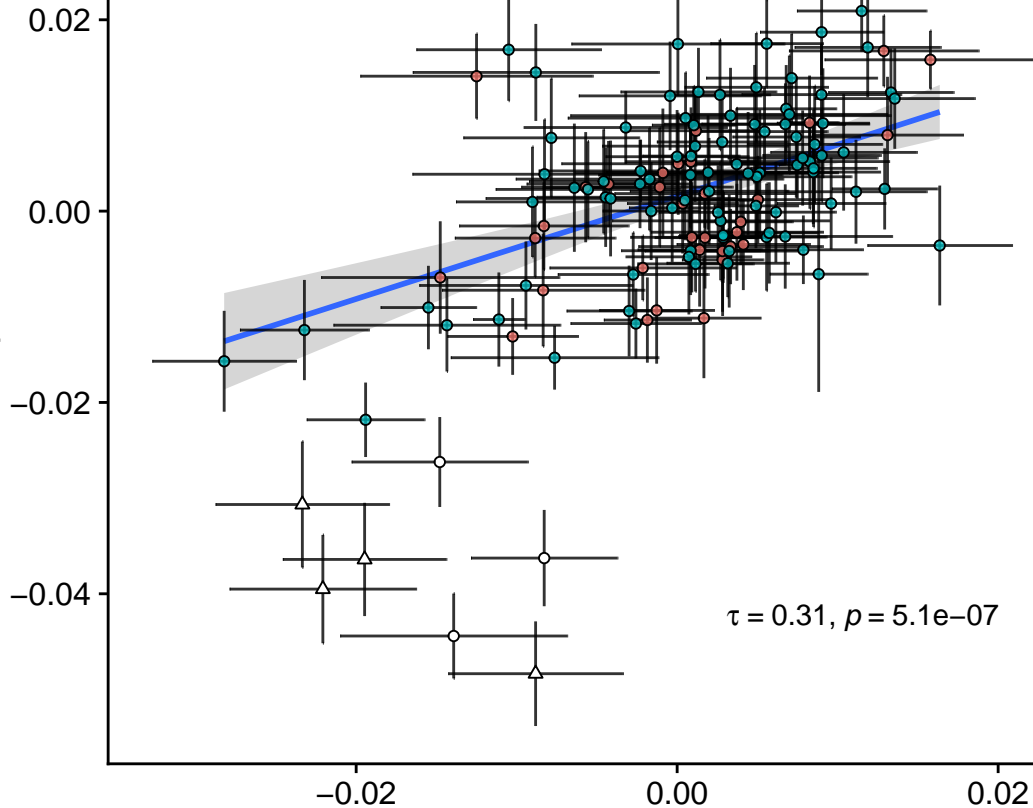

Supplement: Supplemental Information 1 — Triangles signify the four lines whose change in growth rate fell more than −2.5 standard deviations below the mean and were excluded from the analysis. The white triangles and circles signify lines that were unable to grow with galactose as a carbon source. Colored points with grey error bars represent line means and standard error of the mean (calculated from 11 replicate fitness assays). [file peerj-11-16547-s001.pdf]
